# EGF-induced activation of Akt results in mTOR-dependent p70S6 kinase phosphorylation and inhibition of HC11 cell lactogenic differentiation

**DOI:** 10.1186/1471-2121-7-34

**Published:** 2006-09-19

**Authors:** Traci Galbaugh, Maria Grazia Cerrito, Cynthia C Jose, Mary Lou Cutler

**Affiliations:** 1 Department of Pathology, United States Military Cancer Institute, Uniformed Services University of the Health Sciences, Bethesda, MD 20814, USA

## Abstract

**Background:**

HC11 mouse mammary epithelial cells differentiate in response to lactogenic hormone resulting in expression of milk proteins including β-casein. Previous studies have shown that epidermal growth factor (EGF) blocks differentiation not only through activation of the Ras/Mek/Erk pathway but also implicated phosphatidylinositol-3-kinase (PI-3-kinase) signaling. The current study analyzes the mechanism of the PI-3-kinase pathway in an EGF-induced block of HC11 lactogenic differentiation.

**Results:**

HC11 and HC11-luci cells, which contain luciferase gene under the control of a β-casein promotor, were treated with specific chemical inhibitors of signal transduction pathways or transiently infected/transfected with vectors encoding dominant negative-Akt (DN-Akt) or conditionally active-Akt (CA-Akt). The expression of CA-Akt inhibited lactogenic differentiation of HC11 cells, and the infection with DN-Akt adenovirus enhanced β-casein transcription and rescued β-casein promotor-regulated luciferase activity in the presence of EGF. Treatment of cells with Rapamycin, an inhibitor of mTOR, blocked the effects of EGF on β-casein promotor driven luciferase activity as effectively as PI-3-kinase inhibitors. While expression of CA-Akt caused a constitutive activation of p70S6 kinase (p70S6K) in HC11 cells, the inhibition of either PI-3-kinase or mTOR abolished the activation of p70S6K by EGF. The activation of p70S6K by insulin or EGF resulted in the phosphorylation of ribosomal protein S6 (RPS6), elongation initiation factor 4E (elF4E) and 4E binding protein1 (4E-BP1). But lower levels of PI-3-K and mTOR inhibitors were required to block insulin-induced phosphorylation of RPS6 than EGF-induced phosphorylation, and insulin-induced phosphorylation of elF4E and 4E-BP1 was not completely mTOR dependent suggesting some diversity of signaling for EGF and insulin. In HC11 cells undergoing lactogenic differentiation the phosphorylation of p70S6K completely diminished by 12 hours, and this was partly attributable to dexamethasone, a component of lactogenic hormone mix. However, p70S6K phosphorylation persisted in the presence of lactogenic hormone and EGF, but the activation could be blocked by a PI-3-kinase inhibitor.

**Conclusion:**

PI-3-kinase signaling contributes to the EGF block of lactogenic differentiation via Akt and p70S6K. The EGF-induced activation of PI-3-kinase-Akt-mTOR regulates phosphorylation of molecules including ribosomal protein S6, eIF4E and 4E-BP1 that influence translational control in HC11 cells undergoing lactogenic differentiation.

## Background

HC11 mouse mammary epithelial cells have been widely used as an *in vitro *model of mammary gland epithelial cell differentiation. The HC11 cell line preserves important features of mammary epithelial cell lactogenic differentiation; it was clonally derived from the COMMA-1D cells, a line immortalized from mammary tissue of a pregnant BALB/c mouse [1,2]. The HC11 cells are non-tumorigenic, display a normal epithelial phenotype, and the injection of HC11 cells into the cleared fat pad of BALB/c mice exhibited normal ductal and alveolar-like structures [1,3]. HC11 mammary epithelial cell lactogenic differentiation can be initiated in culture following the growth to confluence and deposition of extracellular matrix in the presence of epidermal growth factor (EGF), subsequent removal of EGF from the culture and the addition of lactogenic hormone mix, DIP (dexamethasone, insulin, and prolactin); upon differentiation HC11 cells express specific milk proteins including β-casein [1]. Moreover, during lactogenic differentiation in culture the HC11 cells undergo phenotypic transformation to "mammospheres", enlarged domed structures with a lumen [4-6].

HC11 cells express receptor tyrosine kinases of various subclasses [7,8], and the addition of specific mitogens e.g. EGF or the presence of oncogenes, including activated Ras, inhibit lactogenic differentiation [6,8-11]. Several signaling mechanisms have been shown to facilitate the EGF-induced block of lactogenic differentiation. The two key pathways implicated in HC11 cells are Ras/Raf/Mek/Erk and phosphatidylinositol-3-kinase (PI-3-kinase) pathways [6,8,10,12]. Our previous study demonstrated that DN-Ras expression blocked EGF-induced inhibition of HC11 cell lactogenic differentiation via inhibition of Raf/Mek/Erk signaling and enhanced Stat5 phosphorylation [6]. However, the activation of PI-3-kinase by EGF was largely independent of Ras in these cells, but it did contribute to inhibition of lactogenesis.

The PI-3-kinases are a ubiquitously expressed lipid kinase family that plays a key role in cellular proliferation, growth and survival. PI-3-kinase was initially purified and cloned as a heterodimeric complex consisting of an 110 kDa catalytic subunit and an 85 kDa regulatory/adaptor subunit [13]. Recent reviews of the PI-3-kinase pathway describe its activation and activity [14,15]. The Class I PI-3-kinases [16] are activated following either binding of the p110 subunit to activated Ras [17,18] or binding of the SH2 domains of the p85 adaptor protein to phosphotyrosine residues of the EGF receptor [14]. PI-3-kinase translocates from the cytosol to the membrane where it phosphorylates the 3'-OH position of the inositol ring of substrates including phosphatidylinositol-4, 5-bisphosphate. This phosphorylation directs the membrane localization of 3-phosphoinositide-dependent kinase 1 (PDK1) through its pleckstrin homology (PH) domain resulting in the autophosphorylation of PDK1 and phosphorylation of Akt at Thr 308. Maximal activation of Akt kinase activity requires Ser 473 phosphorylation by a kinase that has yet to be completely characterized and is referred to as PDK2 [19]. There are numerous known Akt substrates including GSK3β, FKHR1 and IKK, and Akt controls aspects of cell survival as well as cell growth and division by phosphorylating these key regulators [20-27].

The activation of Akt can link mitogenic signaling with nutrient sensing pathways that regulate protein synthesis and cell size via a pathway that includes TSC2/tuberin, the GTPase RHEB and the serine-threonine kinase mammalian target of rapamycin, mTOR [28-31]. The activation of mTOR leads to mTOR-initiated phosphorylation of the translation regulators p70S6 kinase and eukaryotic translation initiation factor 4E binding protein 1 (4E-BP1) [32].

The PI-3-kinase and Akt signal transduction pathway contributes to mammary carcinogenesis and resistance of tumors to chemotherapy as a result of mutation and amplification of component members [33-38]. In addition, the control of Akt activity is important in maintaining normal polarized mammary architecture [39-41]. Hence, we examined the importance of the PI-3-kinase pathway in HC11 undergoing lactogenic differentiation. We determined that ectopic expression of conditionally active-Akt blocks lactogenic differentiation and that inhibiting PI-3-kinase, Akt, or mTOR rescues the EGF-induced block of lactogenic differentiation in HC11 mammary epithelial cells. Our data indicate that EGF stimulation activates Akt and subsequently p70S6 kinase, RPS6, eIF4E and 4E-BP1 via PI-3-kinase/Akt dependent mechanisms in HC11 cells. Therefore, activation of PI-3-kinase in HC11 mammary epithelial cells may regulate changes in translational control of proteins that influence the ability of lactogenic hormone to induce differentiation.

## Results

### EGF blocks HC11 lactogenic differentiation via Mek/Erk and PI-3-K dependent pathways

Recent publications from our lab and others [6,12,42] suggest that PI-3-kinase plays a key role in mammary epithelial cell lactogenic differentiation. The present study addresses the mechanism by which PI-3-kinase blocks HC11 mammary epithelial cell lactogenic differentiation. Several parameters defining HC11 mammary epithelial cell differentiation were examined to follow the effects of signal transduction pathways on the differentiation process. The markers include β-casein synthesis and mammosphere formation [1,4,43-45]. Two related cell lines were employed in the study: HC11 mammary epithelial cells and HC11-luci cells which contain a luciferase gene under the control of a β-casein promotor.

**Figure 1:**
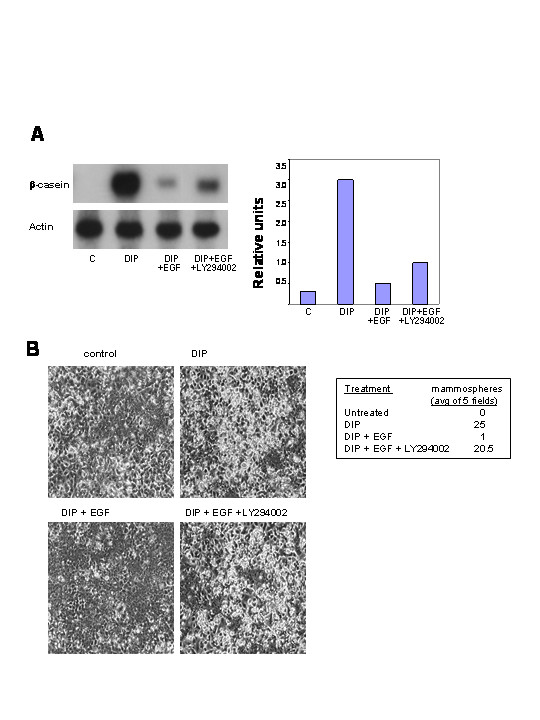
The effect of the PI-3-kinase inhibitor, LY294002 on epidermal growth factor (EGF) disruption of differentiation. **A**. HC11 cells were induced to differentiate in DIP-induction media with and without EGF (10 ng/ml) and LY294002 (5 μM) for 48 hours. β-casein induction was determined via northern blot and was normalized to β-actin. **B**. HC11 cells were grown to confluence as stated above and induced to differentiate in DIP-induction media with and without EGF (10 ng/ml) and LY294002 (10 μM). Cells were photographed at 96 hours post-induction. The number of mammospheres per field is reported: this was determined by counting the number of mammospheres per low power field and determining the mean of five fields.

EGF stimulation of HC11 cells activates PI-3-kinase signaling as well as other pathways, and the results from our previous study determined that EGF blocked activation of a β-casein promotor-luciferase activity following induction of lactogenic differentiation via both Mek/Erk and PI-3-kinase dependent mechanisms [6]. The results in figure 1 confirm and expand those findings using an inhibitor of PI-3-kinase activity. β-casein RNA transcription was examined by northern blotting following stimulation of HC11 cells with the lactogenic hormone mix, DIP, in the presence and absence of EGF and LY294002. EGF blocked lactogenic hormone induced β-casein transcription and the addition of the PI-3-kinase inhibitor, LY294002, partially rescued β-casein transcription (figure 1A). However, the addition of PI-3-kinase inhibitors LY294002 or wortmannin in the absence of EGF reduced all markers of lactogenic differentiation (data not shown), indicating that survival signaling from this pathway was essential for HC11 differentiation to proceed.

Mammosphere formation is another important marker of HC11 lactogenic differentiation. HC11 cells were induced to differentiate in DIP-induction media with or without EGF and LY294002. The cells were observed and photographed at 96 hours post-induction. EGF blocked the formation of mammospheres and LY294002 rescued the EGF block of mammosphere formation (figure 1B). This suggested that PI-3-kinase activation was an important component in the EGF-induced block of phenotypic lactogenic differentiation.

### Constitutive activation of Akt-1 blocks lactogenic differentiation and the expression of dominant negative-Akt enhances differentiation in HC11 cells

**Figure 2:**
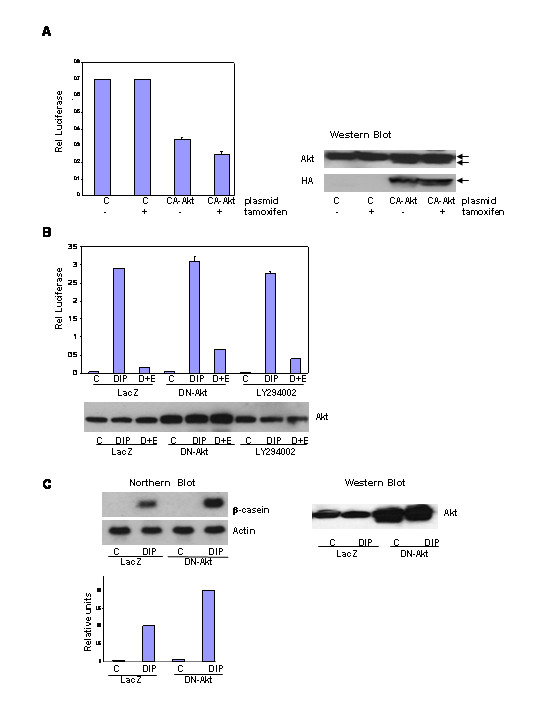
The effect of conditionally active-Akt1 and dominant negative-Akt1 on HC11 differentiation. **A**. The HC11-luci cells transiently transfected with either a conditionally active-Akt-1 (CA-Akt) or a control vector (pCDNA3.1). At 24 hours the cells were incubated in DIP-induction media with or without tamoxifen (1 μM). Luciferase activity was determined 48 hours post-induction and was normalized to protein concentration. Expression of Akt and HA-CA-Akt was determined via western blot. Lanes were loaded with equal amounts of protein (117.5 μg). **B**. The effect of dominant negative-Akt1 (DN-Akt) adenoviral infection on EGF disruption of differentiation. HC11-luci cells were infected with either a DN-Akt1 or control (LacZ) adenovirus. Cells were changed to DIP-induction media the next day and lysates were harvested 48 hours post-induction for β-casein promotor luciferase activity. The results were compared to cultures exposed to DIP plus LY294002 (5 μM). Expression of Akt was determined via western blot analysis and gels were loaded with equal amount of protein (120 μg). **C**: HC11 cells were infected with either a DN-Akt1 or control (LacZ) adenovirus. RNA was harvested 48 hours post-induction for analysis of β-casein RNA expression by northern blot. Expression of Akt was determined via western blot analysis and gels were loaded with equal amount of protein (120 μg).

The activation of Akt is a major outcome of PI-3-kinase stimulation. Hence, the role of Akt in regulating HC11 lactogenic differentiation was examined. Transient transfection of a plasmid encoding a HA-tagged conditionally active-Akt-1 (CA-Akt1) gene was used to assess the ability of the activated Akt pathway to block lactogenic differentiation via inhibition of β-casein promotor luciferase activity [46]. HC11-luci cells were transiently transfected with either a plasmid encoding a HA-tagged conditionally active-Akt1 or a control vector. Western blotting of transfected cell lysates revealed that the HA-tagged conditionally active-Akt1 was expressed at levels equal to the endogenous Akt protein (figure 2A). The cells were induced to differentiate with DIP in the presence of 4-hydroxy-tamoxifen to activate the HA-tagged conditionally active-Akt1, and luciferase activity was determined 48 hours after induction. Expression of the conditionally active-Akt1 significantly decreased luciferase activity compared to the control vector and the addition of tamoxifen slightly reduced the luciferase activity in CA-Akt1 transfected cells (figure 2A). This indicated that the CA-Akt1 was not completely responsive to 4-hydroxy-tamoxifen under these conditions but that there was sufficient activity from the protein to activate PI-3-kinase signaling above that in control cells. The results in figure 4D confirm elevated activation of the pathway.

Infection with a replication defective adenovirus encoding a dominant negative-Akt1 (DN-Akt) containing mutations at both the active site and regulatory serine phosphorylation sites [47] was used to further assess the role of the Akt pathway in blocking lactogenic differentiation. HC11 and HC11-luci cells were grown to 90% confluence and infected with a dominant negative-Akt1 or a control adenovirus. At 24 hours post infection the cells were induced to differentiate in the presence or absence of EGF and then harvested 48 hours later. The amount of DN-Akt was assayed by western blotting and the influence of DN-Akt on the β-casein promotor luciferase activity was determined (figure 2B). In the absence of EGF, infection with the DN-Akt adenovirus did not affect the DIP-induced promotor activity, but DN-Akt partially rescued the EGF-induced inhibition of β-casein promotor luciferase activity compared to LacZ vector control. In addition, the rescue of luciferase activity was greater in the DN-Akt infected cells than in LY294002 treated cells when cells were stimulated with DIP in the presence of EGF.

The effect of DN-Akt on β-casein RNA expression in HC11 cells treated with lactogenic hormone was assessed (figure 2C). Infection with the DN-Akt adenovirus doubled β-casein RNA expression in the HC11 cell line compared to vector control infected cells. Because the expression of conditionally active-Akt1 blocked lactogenic differentiation and dominant negative-Akt1 enhanced lactogenic differentiation, we conclude that Akt activity can contribute to the regulation of lactogenic differentiation in HC11 cells.

### EGF activates p38 Kinase, Jnk and p70S6 Kinase via PI-3-K and mTOR dependent mechanisms in HC11 mammary epithelial cells

**Figure 3:**
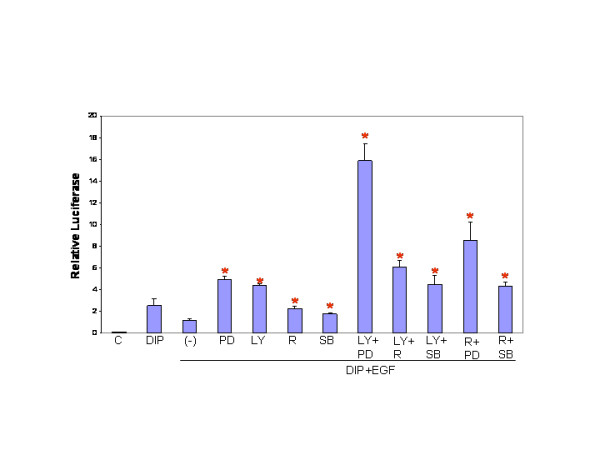
The effect of signal transduction inhibitors on EGF-induced inhibition of lactogenic differentiation. HC11-luci cells were induced to differentiate in DIP-induction media in the presence or absence of EGF (10 ng/ml). Inhibitors were added alone or in combination at the time of induction (LY294002 10 μM, SB203580 10 μM, Rapamycin 100 nM, PD98059 20 μM). Luciferase activity was determined 48 hours post-induction and normalized to protein concentration. *These values represent statistically significant difference (*P *value .0001) from the DIP+EGF.

Both Akt1 and p38MapK have been identified as a potential downstream targets of EGF signaling in mammary epithelial cells. In addition, Akt stimulates activation of mTOR. The effect of blocking PI-3-kinase pathway, including mTOR and the stress kinase pathways, on EGF-induced inhibition of lactogenic differentiation was determined in HC11-luci cells. Inhibitors of Mek, PI-3-kinase and p38 kinase as well as Rapamycin, an mTOR inhibitor, were added to HC11-luci cells in DIP-induction media in the presence of EGF. Luciferase activity was measured 48 hours post-induction and normalized to protein concentration (figure 3). As expected the addition of EGF to the DIP induction media resulted in inhibition of luciferase activity, and each inhibitor alone significantly restored the β-casein promotor activity compared to DIP plus EGF. In combination analyses it appeared that PD98059, the Mek-Erk inhibitor, produced synergistic effects with LY294002 and Rapamycin. However, combinations of LY294002 with Rapamycin and SB203580 produced additive or less than additive responses. This was also the case for a combination of Rapamycin with SB203580. These results demonstrate that the EGF-induced disruption of lactogenic differentiation proceeds by blocking both the Ras-Raf-Mek-Erk pathway and the PI-3-kinase pathway. In addition, the results suggest that EGF-induced activation of mTOR and p38 are both dependent on PI-3-kinase signaling in HC11 cells (figure 3). It should be noted that the increase in luciferase activity detected in inhibitor-treated cells is specific to recovery of activity blocked by EGF. The treatment of HC11-luci cells with high levels of PI-3-kinase or mTOR inhibitors in the absence of EGF reduced cell viability and thereby decreased lactogenic differentiation (data not shown).

**Figure 4:**
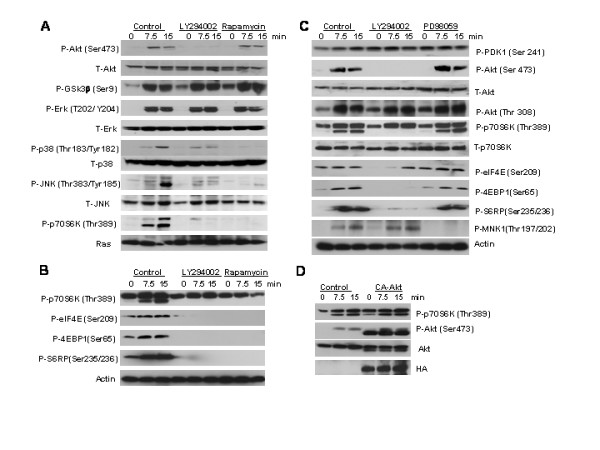
The effect of PI-3-kinase and mTOR inhibitors on signal transduction pathways in HC11 cells. HC11 cells were serum starved overnight and incubated four hours with indicated inhibitors prior to re-stimulation with EGF (100 ng/ml) for times indicated. Lysates were harvested and analyzed by western blotting using antibodies specific for phosphorylated and non-phosphorylated forms of the indicated proteins. **A**. Cells treated with LY294002 (10 μM) and Rapamycin (50 nM) **B**. Cells treated with LY294002 (10 μM) and Rapamycin (50 nM) **C**. Cells treated with LY294002 (10 μM) and PD98059 (20 μM). **D**. The effect of conditionally activated-Akt1 on the Akt and p70S6K signal transduction pathway in HC11 cells. The HC11 cells were transiently transfected with either a conditionally active-Akt1 (CA-Akt) or a control vector. At 24 hours the cells were serum starved overnight in 4-hydroxy tamoxifen (1 μM). The next day the cells were re-stimulation with EGF (100 ng/ml) for times indicated. Lysates were harvested and analyzed by western blotting using antibodies specific for phosphorylated and non-phosphorylated forms of the indicated proteins.

HC11 cells were examined to more fully characterize the effect of PI-3-kinase and mTOR inhibitors on several signal transduction pathways induced by EGF. HC11 cells were serum starved in the absence of EGF and incubated for 4 hours with LY294002 or Rapamycin prior to stimulation with EGF. The cell lysates were harvested following EGF stimulation and analyzed by western blotting for expression and phosphorylation of Akt, Gsk3β, p70S6 kinase and the Map kinases Erk, Jnk and p38. The PI-3-kinase inhibitor completely blocked the phosphorylation and subsequent activation of Akt on serine 473 and p70S6 kinase on threonine 389 and partially blocked the phosphorylation and activation of p38 and Jnk (figure 4A). The mTOR inhibitor Rapapmycin completely blocked the activation of p38, Jnk and p70S6 kinase (figure 4A). However, neither inhibitor blocked the activation of Erk1. The data demonstrate that EGF-induced activation of p38, Jnk and p70S6 kinase in HC11 cells is both PI-3-kinase and mTOR dependent. Because the addition of LY294002 to either Rapamycin or SB203580 did not increase their ability to block effects of EGF, it suggests that blocking PI-3-kinase inhibits p38 and mTOR in HC11 cells. Because the combination of the PI-3-kinase and Mek/Erk inhibitors synergistically increased β-casein promotor luciferase activity (figure 3) and because neither LY294002 nor Rapamycin affects EGF-induced Erk activation (figure 4A), we conclude that the PI-3-kinase and Mek/Erk signaling pathways are independent and synergistic in their ability to block lactogenic differentiation in HC11 cells.

### EGF stimulation results in phosphorylation of ribosomal protein S6, elongation initiation factor 4E, eIF4E-binding protein 1 via PI-3-kinase/mTOR dependent mechanisms

**Figure 5:**
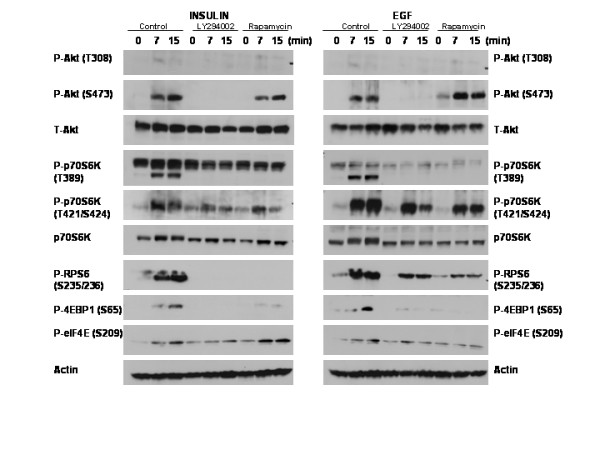
The effect of PI-3-kinase and mTOR inhibitors on insulin and EGF signal transduction pathway in HC11 cells. HC11 cells were serum starved overnight in the absence of insulin and the next day the cells were incubated four hours with LY294002 (5 μM) or Rapamycin (25 nM) prior to re-stimulation with insulin (5 μg/ml) or EGF (100 ng/ml) for times indicated. Lysates were harvested and analyzed by western blotting using antibodies specific for phosphorylated and non-phosphorylated forms of the indicated proteins.

The Akt/mTOR/p70S6 kinase pathway regulates cell growth and proliferation via the regulation of protein synthesis [32]. To elucidate the potential role of PI-3-kinase in HC11 cell protein synthesis we investigated the activation state of potential substrates of p70S6 kinase following EGF stimulation. HC11 cells were serum starved in the absence of EGF and incubated with LY294002, Rapamycin or PD98059 prior to stimulation with EGF. The cell lysates were harvested and analyzed by western blotting using antibodies specific for phosphorylated and non-phosphorylated forms of the indicated proteins. The PI-3-kinase and mTOR inhibitors blocked the phosphorylation of elongation initiation factor 4E (eIF4E) on serine 209, eIF4E-binding protein 1 (4E-BP1) on serine 65, as well as ribosomal protein S6 (RPS6) at Ser235/236 (figure 4B). The Mek/Erk inhibitor blocked the phosphorylation of Mnk1 at Thr197/202 (figure 5A), an event that is known to be Mek/Erk dependent [48]. However, phosphorylation of eIF4E was not affected by PD98059 treatment and the subsequent inhibition of Mnk1, but it was prevented by Rapamycin which blocks p70S6 kinase activation (figure 4B, 4C). This indicates that eIF4E phosphorylation was due to p70S6 kinase and not Mnk1.

The ability of a conditionally active-Akt to activate p70S6 kinase was tested (figure 4D). HC11 cells were transfected with CA-Akt or a vector control plasmid. The expression of conditionally active-Akt in presence of tamoxifen resulted in constitutive activation of p70S6 kinase. Therefore, both EGF stimulation and constitutive Akt can activate p70S6 kinase. Hence, the evidence suggests that one mechanism by which EGF-induced PI-3-kinase activation prevents lactogenic differentiation in HC11 mammary epithelial cells may involve the Akt-dependent activation of p70S6 kinase, and the subsequent phosphorylation of RPS6, eIF4E, and 4E-BP1.

### The role of insulin signal to the PI-3-kinase and mTOR in HC11 cells

Because the growth media, the differentiation media and the starvation media used in the above experiments contained insulin, the results addressed the role of the PI-3-kinase pathway in transmitting EGF-induced signals to Akt, mTOR and p70S6 kinase without considering the potential of insulin to activate the same pathways. To address this question HC11 cells were starved of insulin as well as serum and growth factor, then stimulated with either insulin or EGF in the presence of low levels of PI-3-kinase or mTOR inhibitors. The results in figure 5 detect differences in the p70S6 kinase phosphorylation and kinase activity toward RPS6 that was dictated by the stimulatory agent. Insulin stimulation of Akt (Thr 308 and Ser 473) was PI-3-kinase-dependent, and phosphorylation of p70S6 kinase (Thr389) was PI-3-kinase and mTOR-dependent. Insulin stimulation resulted in PI-3-kinase-and mTOR-dependent RPS6 phosphorylation. In contrast, the stimulation of RPS6 phosphorylation by EGF was partially independent of PI-3-kinase-and mTOR pathways. This additional RPS6 phosphorylation correlated with elevated p70S6 kinase phosphorylation at Thr 421 and Ser 424, the autoinhibitory site reported to contribute to its activity *in vivo *[49]. Because higher levels of PI-3-kinase and mTOR inhibitors completely eliminated this signal (figure 4B and 4C), it appears that EGF requires Akt and mTOR to activate p70S6 kinase and that residual low level activity of p70S6 kinase can be enhanced by EGF-dependent phosphorylation at Thr 421 and Ser 424. Hence, we conclude that both insulin and EGF stimulate the PI-3-kinase-Akt-mTOR-p70S6 kinase pathway, but that EGF modulates p70S6 kinase activity in a manner not activated by insulin.

In addition, differences in the phosphorylation of 4E-BP1 and elF4E were detected in response to insulin and EGF (figure 5). There is mTOR-independent 4E-BP1 and elF4E phosphorylation in response to insulin that is not detected with EGF, suggesting that insulin stimulation of these pathways may be different from that seen with EGF i.e. that insulin signaling may phosphorylate these substrates via a pathway other than mTOR.

### Dexamethasone contributes to the inhibition of p70S6 kinase during lactogenic differentiation of HC11 cells

**Figure 6:**
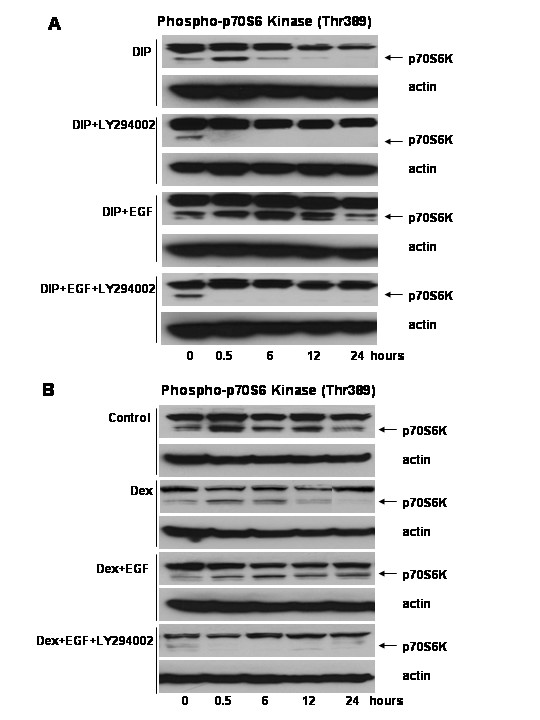
The long-term activation of the p70S6 kinase pathway in HC11 cells. **A: **HC11 cells were induced to differentiate in DIP-induction media with and without EGF (10 μg/ml) and LY294002 (10 μM) for times up to 24 hours. Lysates were harvested and analyzed by western blotting using antibodies specific for phosphorylated p70S6 kinase **B: **HC11 cells were exposed to dexamethasone (1 μM) with and without EGF (10 μM) and LY294002 (10 μM) for times up to 24 hours. Lysates were harvested and analyzed by western blotting using antibodies specific for phosphorylated p70S6 kinase or actin. The arrows indicate the position of the phospho-p70S6 kinase band.

The studies described above addressed short-term stimulation of cells with EGF. Next, the long-term activation of signal transduction pathways dependent on PI-3-kinase stimulation was examined in HC11 cells (figure 6A). HC11 cells were treated with lactogenic hormone mix in the presence or absence of EGF and LY294002 for times up to 24 hours. Cell lysates were analyzed by western blotting for phosphorylation of p70S6 kinase. In the HC11 cells stimulated with the lactogenic hormone mix DIP the activation of p70S6 kinase on threonine 389 completely diminished by approximately 12 hours, whereas in the cells stimulated with DIP in the presence of EGF the activation of p70S6 kinase persisted for 24 hours. In contrast, the cells exposed to DIP and EGF with LY294002 showed no p70S6 kinase activation at any time point after induction. These results suggest that blocking the PI-3-kinase pathway at the time of DIP-induction enhanced differentiation via a similar mechanism to that described above in short term assays, *i.e*. inactivation of Akt/mTOR/p70S6 kinase.

Dexamethasone is a component of the lactogenic hormone mix, DIP. Because dexamethasone can inhibit p70S6 kinase phosphorylation and protein synthesis [50], we investigated the ability of dexamethasone alone to inhibit the phosphorylation of p70S6 kinase (figure 6B). HC11 cells were exposed to dexamethasone in the presence or absence of EGF and LY294002 for times up to 24 hours. The lysates were analyzed by western blotting for phosphorylation of p70S6 kinase. In the HC11 cells treated with dexamethasone the phosphorylation of p70S6 kinase decreased during the first 12 hours, while cells exposed to a combination of dexamethasone and EGF showed p70S6 kinase phosphorylation through 24 hours. The cells treated with dexamethasone and EGF plus LY294002 exhibited no p70S6 kinase activation at any time point after induction. These results suggest that dexamethasone inhibits p70S6 kinase phosphorylaton and that the presence of EGF overcomes the inhibitory effect of dexamethasone on this pathway.

## Discussion

Mammary gland development can be divided into seven stages: embryonic, postnatal, juvenile, puberty, pregnancy, lactation, and involution. One of the leading risk factors for breast cancer is nullparity [51]. Hence, the delineation of factors that regulate lactogenesis (terminal differentiation) is important in understanding the etiology of breast cancer.

Excess activation of signaling pathways downstream of the epidermal growth factor receptor, ErbB1, has been directly linked to breast cancer development and chemotherapeutic resistance [52]. While EGF is required for normal mammary epithelial cell proliferation, it has been shown to inhibit lactogenic differentiation of HC11 mammary epithelial cells both *in vitro *and *in vivo*, concomitant with stimulation of the Ras/Mek/Erk and the PI-3-kinase pathways [6,8,9,12]. The PI-3-kinase pathway is important in tumorigenesis in several ways. Aberrant PI-3-kinase activation has been demonstrated to promote both proliferation and survival of transformed cells, including those exhibiting EGF-dependent transformation. The mutation and deregulation of PI-3-kinase pathway components has recently been linked to a number of human malignancies [33-38] and breast cancer associated mutations of the p110 catalytic subunit of PI-3-kinase were oncogenic when tested in immortalized mammary epithelial cells [53]. Elevated Akt levels have been found in breast, ovarian, colon and thyroid cancers [54,55].

The data reported here confirm and extend our earlier results indicating that PI-3-kinase inhibitors rescue the EGF-induced block of β-casein promotor-regulated luciferase activity, β-casein transcription and mammosphere formation in lactogen-treated HC11 cells. Furthermore, the expression of a conditionally active-Akt1 blocked lactogenic differentiation, whereas dominant negative-Akt1 enhanced it. These results indicate that EGF blocks HC11 lactogenic differentiation in part via a PI-3-kinase/Akt dependent mechanism. In addition, our data indicate that in HC11 cells PI-3-kinase regulated the EGF-dependent transcription of cyclin D1 and osteopontin (OPN) (Wang, Galbaugh, and Cutler, unpublished observation), both of which are regulated by the PI-3-kinase pathway in tumor cells [56,57]. However, PI-3-kinase inhibition did not entirely prevent the EGF-induced reduction in transcription of differentiation specific target genes. For example, EGF blocks transcription of prolactin-induced protein, PIP, via the Mek/Erk and not PI-3-kinase pathways (Wang, Galbaugh and Cutler, unpublished data). Consequently, we conclude that the involvement of the PI-3-kinase pathway in blocking lactogenic differentiation is partly independent of Stat5-induced transcriptional changes.

The inhibitory effect of PI-3-kinase on β-casein transcription and β-casein promotor luciferase activity is likely through combined mechanisms involving signals mediated by prolactin and dexamethasone. Dexamethasone can play a role in inhibiting the phosphorylation of p70S6 kinase thereby decreasing protein synthesis [50]. Our study reveals that dexamethasone inhibits the phosphorylation of p70S6 kinase in HC11 cells. This suggests a role for dexamethasone in lactogenic hormone-induced differentiation in addition to its role in activating glucocorticoid receptor, which acts synergistically with Stat5 to initiate β-casein transcription [58-60]. PI-3-kinase mediated translational control influences differentiation in erythroid precursers. Stem cell factor delays differentiation of erythroid precursers in part by activating PI-3-kinase pathway resulting in 4E-BP1 phosphorylation and the subsequent recruitment of growth-specific mRNAs into polysomes [61]; and ectopic expression of eIF4E in these cells has the same effect [62]. Our work has not identified specific protein targets whose synthesis is translationally regulated by the PI-3-kinase/Akt/mTOR pathway in HC11 cells. However, a recent study demonstrated that ErbB2 increases the synthesis of the vascular endothelial growth factor (VEGF) protein via the activation of mTOR and p70S6K in human breast cancer cells [63]. This finding suggests that it may be essential to down regulate VEGF or other growth factors in order for lactogenic differentiation to proceed. Also, SOCS-1 can be translationally repressed via a cap-dependent mechanism [64], suggesting that another effect of activation of PI-3-kinase pathway may be the elevation of SOCS-1 and inhibition of prolactin-induced Jak-Stat signaling.

Through the use of chemical inhibitors, alone or in combination, our data revealed that the PI-3-kinase and Mek/Erk signaling pathways are independent and synergistic in their block of HC11 lactogenic differentiation. We determined that EGF activates phosphorylation of Akt, mTOR, p70S6 kinase, ribosomal protein S6, eIF4E and 4E-BP1 in a PI-3-kinase dependent manner, and PI-3-kinase activation may prevent lactogenic differentiation in HC11 mammary epithelial cells by regulating the synthesis of proteins.

While several studies have suggested that Erk activation can be regulated through the PI-3-kinase pathway [65,66] our data demonstrated that EGF stimulation of Erk activation in HC11 mammary epithelial cells was not altered by blocking PI-3-kinase signaling with LY294002. In addition, our previous work revealed that PI-3-kinase activation by EGF receptor proceeded without requiring Ras activation [6]. A report by Bailey et al. demonstrated that low level activation of Akt by prolactin stimulation blocked the inhibitory effects of exogenous TGFβ on HC11 cells [42]. Our study examined the effects of stronger Akt activation by mitogen rather than by TGFβ, which induces apoptosis in HC11 cells. While no previous studies have addressed the mechanism by which PI-3-kinase blocks lactogenic differentiation, we demonstrated that the inhibition of PI-3-K, Akt or mTOR blocked the activation of p70S6 kinase and its downstream targets. We also demonstrated that the expression of a conditionally active-Akt1 leads to the constitutive activation of p70S6 kinase. Interestingly, we discovered that PDK1 is constitutively phosphorylated in HC11 cells and this is not blocked by LY294002. While PDK1 has been shown to directly activate p70S6 kinase independently of Akt [67], our results indicate that the activation of p70S6 kinase is dependent on Akt and mTOR in HC11 cells.

The present study enhances our knowledge of HC11 mammary epithelial differentiation in several ways. We demonstrated that Akt activation can inhibit lactogenic hormone induced differentiation in mammary epithelial cells. Two previous studies questioned whether PI-3-kinase activation of Akt in normal mammary epithelial cells is sufficient for cellular transformation [68,69]. Our observation that blocking the activation of PI-3-kinase restored mammosphere formation, which was inhibited by EGF, is in agreement with reports that conditionally active-Akt1 promotes large and misshapen acinar structures in MCF-10A cells [39,40]. However, the results obtained from cell culture experiments are somewhat different from *in vivo *analysis of Akt. Akt is expressed during lactation *in vivo *at a point when levels of other kinases are diminishing [70]. The expression presumably plays a critical function in cell survival at this point in mammary differentiation. The transgenic expression of MMTV-CA-Akt enhanced/temporally extended the expression of β-casein and resulted in more differentiated cells surviving in the tissue during lactation again at the time when other receptor tyrosine kinases were nearly absent [71,72]. Recently Jankiewitz et al. demonstrated that treatment of lactating mice with rapamycin decreased the size of the mammary glands and inhibited HC11 differentiation by blocking lactogenic hormone-induced expression of the transcriptional regulator Id2 [73]. Our HC11 experiments were performed in immortalized HC11 cells grown in the presence of insulin and fetal bovine serum, sources of stimulation for other receptor tyrosine kinases including those required for cell survival. We also found that blocking PI-3-kinase signaling with chemical inhibitors in the absence of additional mitogen decreased HC11 lactogenic differentiation. However, the stimulation of downstream pathways by EGF or CA-Akt was in excess of the normal cell survival signaling and thereby altered cell responses accordingly. Our results indicate that activation of p70S6 kinase under those conditions is detrimental to HC11 lactogenic differentiation. While this study presents a comprehensive investigation of the role that EGF-induced PI-3-kinase and Akt play in HC11 lactogenic differentiation, further studies in animal models will provide a greater understanding of the role of PI-3-kinase and p70S6 kinase on ErbB1 signals during hormonal regulation of the mammary gland.

## Conclusion

Our results indicate that EGF-induced activation of PI-3-kinase results in Akt- and mTOR-dependent-p70S6 kinase phosphorylation in HC11 cells. The EGF-induced activation of PI-3-kinase-Akt-mTOR regulates phosphorylation of molecules including RPS6, eIF4E and 4E-BP1 that influence translational control. The activation of this pathway contributes to the inhibition of HC11 lactogenic differentiation by EGF.

## Methods

### Cell culture and lactogenic hormone induced differentiation

HC11 and HC11-luci mouse mammary epithelial cell lines were a generous gift from Dr. Nancy Hynes [9,74]. The HC11-luci cell line contains a luciferase gene under the control of a β-casein promotor [7,75]. The cells were maintained in growth media: RPMI 1640 medium augmented with 10% fetal bovine serum (FBS), 5 μg/ml Insulin, 10 ng/ml epidermal growth factor (EGF), 10 mM HEPES, Pen-Strep, and 2 mM Glutamine. The technique for lactogenic differentiation of HC11 cells was described previously [6,9,74]. Briefly, HC11 and HC11-luci cells were grown to confluence and maintained 1–3 days in RPMI 1640 growth media. EGF-containing media was removed, cells were rinsed with media containing lacking EGF, and incubated in RPMI differentiation media, referred to as DIP, containing either 1% FBS or 10% FBS, dexamethasone (10^-6 ^M), 5 μg/ml Insulin, and 5 μg/ml ovine prolactin (PRL)(Sigma). The cells were harvested and processed using stated procedures. HC11 differentiation was characterized by mammosphere formation and β-casein transcription. Mammospheres formation was observed up to 96 hours post DIP treatment [4,5,76]. Mammospheres were enumerated by microscope observation and photographed as described previously [6]. The number of mammospheres was determined by counting the number of mammospheres per low power field and determining the mean of five fields. β-casein transcription was assessed via northern blotting. HC11-luci lactogenic differentiation was characterized via β-casein promotor driven luciferase activity.

### Transfection of cells

The HC11 and HC11-luci cells were transiently transfected with either a conditionally active-Akt-1 (myrΔ4-129-ER or referred to CA-Akt in the paper) or a control construct (pCDNA3.1), which were generously provided by Dr. Richard Roth [46]. The conditionally active-Akt-1 was created by attaching a *src*myristoylation signal to the amino terminus of a variant Akt that lacked the PH domain and carried an HA epitope tag at its carboxyl terminus. This was then fused in frame to the hormone-binding domain of a mutant form of the murine estrogen receptor therefore making it responsive to the synthetic steroid 4-hydroxy-tamoxifen [46]. The cells were transfected at 80% confluence in 35 mm wells with 3 μg of plasmid DNA and Gene Juice (Novagen) as recommended by manufacturer.

### Adenovirus propagation, titration and infection

HEK-293 cells (ATCC) used for virus propagation were maintained in DMEM medium augmented with 10% FBS, Pen-Strep, and 2 mM Glutamine. 25 × T-175 flasks of 293 cells were grown to 90% confluence and infected with either a replication defective Lac Z control adenovirus or DN-Akt1 (DN-Akt) adenovirus kindly provided by Dr. Kenneth Walsh [47]. The DN-Akt1 vector contains alanine substitutions at the active site (residue179) as well as both regulatory phosphorylation sites (Thr308, Ser473) and a HA-Tag at its N-terminus [47]. Cells were harvested 48 hours post infection, pelleted and resuspended in PBS. Following four freeze-thaw cycles the virus was purified via a cesium chloride gradient and dialyzed against a buffer containing 10 mM Tris, 2 mM MgCl_2_, 100 mM NaCl and 5% Glycerol. 293 cells were used for titration of the virus: cells were infected with serial dilutions of virus ranging from 10^-2 ^to 10^-8 ^and cytopathic effect (CPE) was assessed at 24 and 48 hours. HC11 and HC11-luci cells were infected with either the Lac-Z control adenovirus or DN-Akt1 adenovirus at MOI of 10. After 5 hours virus was removed, regular growth media was added and cells were incubated 16–24 hours prior to treatment.

### Luciferase assays

The luciferase technique was performed as previously described [6]. Inhibitors were added alone or in combination at the time of induction of lactogenisis at previously determined optimal concentrations (LY294002 10 μM, SB203580 10 μM, Rapamycin 50 nM, PD98059 20 μM). Luciferase activity was assayed 48 hours post-induction using a commercial luciferase kit (Luciferase Assay Systems, Promega) and a Thermolab System luminometer (Acscent FL). Luciferase activity was normalized to protein concentration as determined by BCA assay (Pierce, Rockford, IL). Results were presented as relative units calculated from the mean of three determinations.

### Western blots

HC11 cells were lysed in either RIPA buffer (1% NP40, 0.5% DOC, 0.1% SDS, 150 mM NaCl, 5 mM MgCl_2 _and 25 mM Hepes) or a high salt buffer [60]. Each lysis buffer contained AEBSF (20 μg/ml), aprotinin (5 μg/ml), leupeptin (5 μg/ml), β-glycerol phosphate (100 μM), and NaVAO_4 _(1 mM). For western blots equivalent amounts of protein were separated by SDS-PAGE and transferred to PVDF filters. The filters were blocked in 0.6% Blotto for one hour and then incubated with the appropriate primary antibody for one hour at room temperature or overnight at 4°C on a rocker. Blots were incubated with appropriate HRP-conjugated secondary antibody for 1 hour at room temperature and washed three times for 10 minutes in TBST. Chemiluminescence was detected with either ECL (Amersham) or Supersignal (Peirce) using Classic Blue Sensitive x-ray film (Midwest Scientific) or collection of images on a CCD camera. All blots were quantitated via scanning densitometry (Fuji, Image gauge software). Antibodies include anti-phospho-Akt (Ser 473 and Thr308) and anti-Akt (Cell Signaling Technology), anti-phospho-GSK3β (Cell Signaling Technology), anti-phospho-Erk (Cell signaling Technology), anti-Erk1(Santa Cruz Biotechnology), anti-phospho-p38 (Cell Signaling Technology), anti-p38 (Santa Cruz Biotechnology), anti-phospho-Jnk (Cell Signaling Technology), anti-Jnk (Santa Cruz Biotechnology), anti-phospho-p70S6 kinase (Thr389) or (Thr 421/Ser 424) (Cell Signaling Technology), anti-p70S6 kinase (Cell Signaling Technology), anti-phospho-eIF4E (Ser209) (Cell Signaling Technology), anti-phospho-4E-BP1 (Ser65) (Cell Signaling Technology), anti-phospho-ribosomal protein S6 (Ser235/236) (Cell Signaling Technology), anti-phospho-Mnk1 (Thr197/202), anti-Pan Ras (Calbiochem), anti-β-Actin (clone AC-15) (Sigma) and anti-HA (clone 12CA5) (Roche). Antibodies were used at manufacturer's dilution recommendation.

### Northern blot

Total cell RNA was extracted and 7.5 ug of RNA was separated on a 1% agarose-formaldehyde gel and transferred to a nylon filter. Blots were hybridized with probes for mouseβ-casein and mouse β-Actin as described previously [6].

## Abbreviations

EGF, epidermal growth factor; PI-3-K, phosphatidylinositol-3-kinase; DIP, dexamethasone, insulin and prolactin; PRL, prolactin; MECs, mammary epithelial cells; DN, dominant negative; CA, conditionally active; PIP, prolactin inducible protein; OPN, osteopontin

## Competing interests

The author(s) declare that they have no competing interests.

## Authors' contributions

T.G. carried out the carried out cell culture procedures, growth and sequencing of plasmids and growth and purification of adenovirus, many of the luciferase assays and immunoassays, and drafted the manuscript. M.G.C. participated in the discussion and design of the study and performed preliminary cell culture analyses. C.J. performed RNA isolation, some northern and western blots. M.L.C. conceived of the study, participated in its design and coordination, and prepared the final draft of the manuscript. All authors read and approved the final manuscript.
